# Improving 2-Chlorotrityl Chloride (2-CTC) Resin Activation

**DOI:** 10.3390/mps6050082

**Published:** 2023-09-08

**Authors:** Tanya Román, Gerardo Acosta, Beatriz G. de la Torre, Constanza Cárdenas, Fanny Guzmán, Fernando Albericio

**Affiliations:** 1 Núcleo Biotecnología Curauma, Pontificia Universidad Católica de Valparaíso, Valparaíso 2373223, Chile; tanya.roman.21@gmail.com (T.R.); constanza.cardenas@pucv.cl (C.C.); fanny.guzman@pucv.cl (F.G.); 2 Doctorado en Biotecnología, Pontificia Universidad Católica de Valparaíso, Universidad Técnica Federico Santa María, Valparaíso 2373223, Chile; 3 Department of Organic Chemistry and CIBER-BBN, Networking Centre on Bioengineering, Biomaterials and Nanomedicine, University of Barcelona, 08028 Barcelona, Spain; gerardoacosta@ub.edu; 4 Institute for Advanced Chemistry of Catalonia (IQAC-CSIC), Jordi Girona 18-26, 08034 Barcelona, Spain; 5 KwaZulu-Natal Research Innovation and Sequencing Platform (KRISP), School of Laboratory Medicine and Medical Sciences, College of Health Sciences, University of KwaZulu-Natal, Durban 4041, South Africa; garciadelatorreb@ukzn.ac.za; 6 Peptide Science Laboratory, School of Chemistry and Physics, University of KwaZulu-Natal, Westville, Durban 4000, South Africa

**Keywords:** 2-CTC resin activation, 2-CTC resin reutilization, resin loading, solid-phase peptide synthesis

## Abstract

Used in solid-phase peptide synthesis (SPPS) for peptides with an acid termination, the 2-chlorotrityl chloride (2-CTC) resin is highly susceptible to moisture, leading to reduced resin loading and lower synthetic yields. It is therefore recommended that the resin be activated with thionyl chloride (SOCl_2_) before peptide assembly. Here we present an optimized procedure for resin activation that minimizes the use of SOCl_2_ as the activation reagent and reduces the activation time. Additionally, we demonstrate the feasibility of reusing the 2-CTC resin when following the activation protocol, achieving comparable results to the first usage of the resin. Moreover, we achieved different degrees of resin activation by varying the amount of SOCl_2_. For instance, the use of 2% SOCl_2_ in anhydrous dichloromethane (DCM) allowed up to 44% activation of the resin, thereby making it suitable for the synthesis of longer peptides. Alternatively, employing 25% SOCl_2_ in anhydrous DCM resulted in up to 80% activation with a reaction time of only 5 min in both cases.

## 1. Introduction

The 2-chlorotrityl chloride (2-CTC) polystyrene resin is used in solid-phase peptide synthesis (SPPS) to obtain peptides with an acid termination. Although its initial purpose was to obtain protected peptides for use in convergent synthesis, this resin proved also to be quite useful for synthesizing fully unprotected peptides [1]. 2-CTC resin offers several advantages in the synthetic process. In this regard, it prevents racemization during the incorporation of the first protected amino acid and minimizes diketopiperazine (DKP) formation [1,2]. It has been widely used in SPPS for the preparation of peptides with multiple applications [3,4,5], including cyclic peptides [6,7,8]. Due to its chemical structure, the resin is highly sensitive to moisture, and over time its load capacity tends to decrease, rendering the alcohol [2]. Given that the alcohol is non-reactive, poor substitutions in the incorporation of the first amino acid are obtained. In this context, resin pre-activation guarantees an adequate degree of substitutions. 

In previous works done in our laboratory, the pre-activation was carried out using 50% of SOCl_2_ in DCM [9] and 7.5% of SOCl_2_ in DMF at 38 °C [1]. Both methods may require a fine tuning. The first one due to the large amount of SOCl_2_ used and the second one for using some heating and the resin colour change, which could indicate some chemical modification of the resin. 

In this work, 2-CTC resin was activated considering several factors, namely the concentration of thionyl chloride (SOCl_2_) and the activation time on the one hand, and the presence of water in the activation and incorporation of the first amino acid steps on the other. 

Moisture significantly affected resin activation, causing a decrease in substitution and therefore in synthetic yield. These results thus highlight the importance of using anhydrous DCM.

## 2. Experimental Section

### 2.1. Materials and Reagents

#### 2.1.1. Reactor

A syringe with a polypropylene filter was used as a reactor. It was attached to a vacuum pump for drainage.

#### 2.1.2. Resin

- 2-Chlorotrityl chloride resin (nominal loading 1.59 mEq/g)

#### 2.1.3. Fmoc-Amino Acids-OH

- Fmoc-L-Thr(tBu)-OH
- Fmoc-L-Lys(Boc)-OH
- Fmoc-L-Gly-OH

#### 2.1.4. Synthesis Grade Solvents

- N-Methyl-2-pyrrolidone (NMP)
- Methanol (MeOH)

#### 2.1.5. Reagents to Activate the Resin

- Thionyl chloride (SOCl_2_)
- Diisopropyl-ethylamine (DIEA)
- NaOH
- Anhydrous dichloromethane (anh. DCM)
- Methanol (MeOH)

#### 2.1.6. Other Reagents

- Ethyl 2-cyano-2-(hydroxyimino)acetate (OxymaPure)
- N,N′-diisopropylcarbodiimide (DIC)

The resin and protected amino acids were purchased from Iris Biotech GmbH (Marktredwitz, Germany). The solvent and reagents used to activate the resin were supplied by Merck KGaA (Darmstadt, Germany). OxymaPure and DIC were generous gifts from Luxembourg Bio Technologies (Nes Ziona, Israel).

### 2.2. Preparation of Solutions

Each solution was prepared just before use.

**(A) 50% Activation solution (Sol. A):** 50% *v*/*v* SOCl_2_ in anh. DCM. 10 mL was prepared of Sol. A: For this 5 mL of SOCl_2_ was added in a 25 mL cylinder and filled to 10 mL with anh. DCM.

**(B) 25% Activation solution (Sol. B):** 25% *v*/*v* SOCl_2_ in anh. DCM. 10 mL was prepared of Sol. B: For this 2.5 mL of SOCl_2_ was added in a 25 mL cylinder and filled to 10 mL with anh. DCM.

**(C) 2% Activation solution (Sol. C):** 2% *v*/*v* SOCl_2_ in anh. DCM. 10 mL was prepared of Sol. C: For this 200 μL of SOCl_2_ was added in a 25 mL cylinder and filled to 10 mL with anh. DCM.

**(D) Amino acid incorporation solution:** 3 Eq of protected amino acids in 1 mL of anh. DCM.

### 2.3. Activation Protocol and Incorporation of the First Amino Acid

To determine the variables on the resin load, the following was considered:

1. The effect of SOCl_2_ concentration and the activation step.
2. The effect of moisture that was reflected in the use of anh. DCM with certain percentages of H_2_O added (using MilliQ water) in two different experiments. First, in the activation step, water was added together with SOCl_2_ before incorporating the amino acid, and, second, water was added together with the amino acid coupling solution after activation. 5 and 10%, of H_2_O were added to obtain DCM saturated with H_2_O.

In the first case, solutions A, B, and C from Section 2.1 were used, corresponding to 50, 25, and 2% SOCl_2_ in anh. DCM respectively, and the incorporation of the first protected amino acid was carried out at 5, 20, 40, and 60 min.

To determine the effect of moisture, a solution of 50% *v*/*v* SOCl_2_/anh. DCM was used, with 30 min of activation and 2 h of coupling, adding different percentages of H_2_O to the anh. DCM, as shown in Table 1. H_2_O was added either during the activation or first amino acid incorporation steps. 

#### 2.3.1. Anhydrous DCM

**Figure 1:**
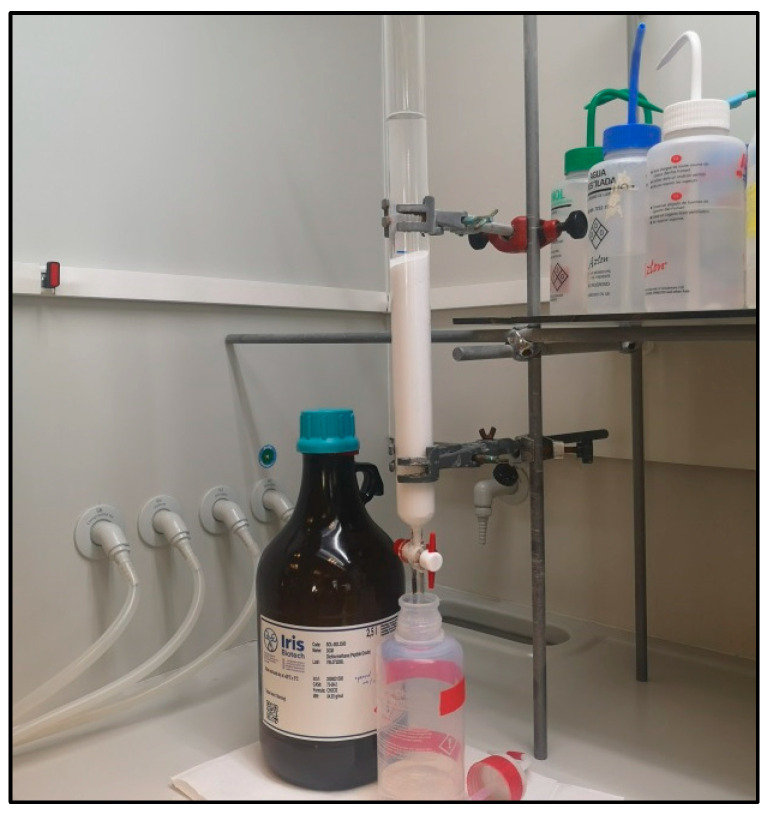
DCM drying process with the use of a basic aluminum oxide column.

To assess the impact of moisture on resin loading, it is crucial to use anh. DCM for both the activation step and the incorporation of the first protected amino acid onto 2-CTC resin. If anh. DCM is not available, it can be dried by passing the solvent through a basic aluminum oxide column (Figure 1). This process helps to ensure the integrity and effectiveness of the resin during the synthesis.

#### 2.3.2. 2-CTC Resin Activation

Weigh 100 mg of resin and divide it into four equal aliquots. Place each aliquot in a separate reactor. Swell the resin by adding 1 mL of anh. DCM to each reactor and allow it to stand for 10 min. Discard the solvent from each reactor. Next, add 1 mL of SOCl_2_/anh. DCM solution (either Solution A, B, or C, as mentioned in Section 2.2) to each reactor. Shake the reactors for 5, 20, 40, or 60 min, ensuring that each duration is assigned to a specific sample. After the activation process, discard the solution from each reactor into a round-bottom flask containing 1 M NaOH. Thoroughly wash each sample with 1 mL of anh. DCM, repeating this step five times. Collect the washings in the flask containing the basic solution.

#### 2.3.3. Amino Acid Coupling

First, weigh out Fmoc-Thr(tBu)-OH (3 Eq.), dissolve it in 1 mL of anh. DCM, and add it to the reactor containing the activated resin. Immediately, add 9 Eq. of DIEA and shake the mixture for 2 h. To cap the non-coupled sites, add 100 µL of MeOH and stir the mixture for another 30 min. Then, discard the solution, and wash the resin with anh. DCM.

#### 2.3.4. Effect of Water

To assess the relevance of the use of anh. DCM, determine the effect of moisture on resin loading for both the activation step and the incorporation of the first protected amino acid. Thus, add H_2_O at 5 and 10% to solution A for the activation step (step 2.3.2, experiments 2 and 3 in Table 1), and 5 and 10% H_2_O to solution D for the incorporation of the first protected amino acid (step 2.3.4, experiments 4 and 5 in Table 1).

### 2.4. Loading Quantification

To determine the resin substitution, remove the Fmoc group from the first amino acid incorporated using 1 mL of 20% piperidine in DMF for 5 min. Collect the drained solvent during this process in a 50 mL volumetric flask. Repeat the deprotection step for another 5 min, and collect the drained solvent in the same 50 mL volumetric flask (Dilution 1). Wash the resin twice with 1 mL of DMF, collecting the drained solvent each time in the same 50 mL volumetric flask, bringing the total volume up to 50 mL with DMF. Take an aliquot (Aliquot) of this solution, ranging between 50 and 500 µL (depending on the amount of deprotected resin), and transfer it to a separate 25 mL volumetric flask (Dilution 2). For example, if you have 100 mg of total resin, add 500 µL of the solution to the flask, and if you have 1 g of total resin, add only 50 µL. Fill up the remaining volume with DMF to reach 25 mL.

The absorbance of the solution is measured at 290 nm, using a quartz cuvette and pure DMF as a blank. The resin load is calculated using Equation (1):

(1) Substitution=(Abs×DF)/(5800×1×g)

Abs = Measured absorbance; DF = Dilution factor *; g = grams of resin used

(2) DF=(Dilution 1×Dilution 2)/(Aliquot)

* Dilution factor, in this case, corresponds to the dilution to which the piperidine solution used is subjected, according to the aliquot taken. For example, if piperidine is added to a 50 mL volume flask and then a 100 µL aliquot is taken and added to a 25 mL volume flask, the dilution factor will be 50 × 25/0.1 = 12,500.

### 2.5. Synthesis of a Model Tripeptide 

To evaluate the performance of the resin under the different humidity conditions, the model tripeptide Fmoc-Gly-Lys(Boc)-Thr(tBu)-OH was synthesized and characterized. using a non-activated resin sample (Experiment 6 in Table 1) and resin samples with and without the effect of humidity in the activation and coupling steps of the first amino acid (Experiment 3, 5 and 1 in Table 1).

### 2.6. Cleavage

To remove the peptide from the resin while preserving its protecting groups, the resin is washed twice with DMF (5 mL/g resin) and twice with DCM (5 mL/g resin). Next, a 1% TFA solution in DCM is prepared and added to the resin (10 mL/g resin), and the mixture is shaken for 1 min and drained over 30 mL of H_2_O, to keep the acid concentration low and prevent the cleavage of the side chain protecting groups. This step is repeated four times. Next, the DCM is evaporated, and the precipitated amino acid in H_2_O is dissolved using 10 mL of ACN. Finally, freezing and lyophilization proceed for 48 h.

### 2.7. Characterization

The characterization of the model tripeptide is carried out by means of high-performance liquid chromatography (HPLC) to evaluate the purity and LC-MS to confirm its identity. 

#### 2.7.1. Reverse-Phase High-Performance Liquid Chromatography (RP-HPLC)

The determination of the percentage of purity of the Fmoc-N-Me-AA-OH is done by RP-HPLC using a 30 to 100% acetonitrile (ACN) gradient in 8 min. Column: XBridge BEH 130, 3.5 μm, 4.5 mm × 100 mm. Solvents: Water with 0.045% of TFA and ACN with 0.036% of TFA. Wavelength Detection: 220 nm. Flow: 1 mL/min. 

#### 2.7.2. Liquid Chromatography-Mass Spectrometry (LC-MS)

The presence of tripeptide is evaluated by LC-MS, showing the peaks for the corresponding [M + H]1+ of each AA, using a 30 to 100% ACN gradient in 3.5 min. Column: XSelect 3.5 μm, 4.6 × 50 mm. Solvent: water 0.1% formic acid and ACN 0.07% formic acid. Wavelength Detection: 220 nm. Flow: 1.6 mL/min.

### 2.8. Statistical Analysis

Two-way standard multiple comparison analysis of variance (ANOVA) is used to compare loadings at different times and different % SOCl_2_. Finally, Tukey’s multiple comparison test is used for pairwise comparison. A *p* < 0.05 is considered statistically significant. The statistical analysis is performed with GraphPad Prism version 9.0 (GraphPad, San Diego, CA, USA).

## 3. Results

### 3.1. Activation Protocol 

**Figure 2:**
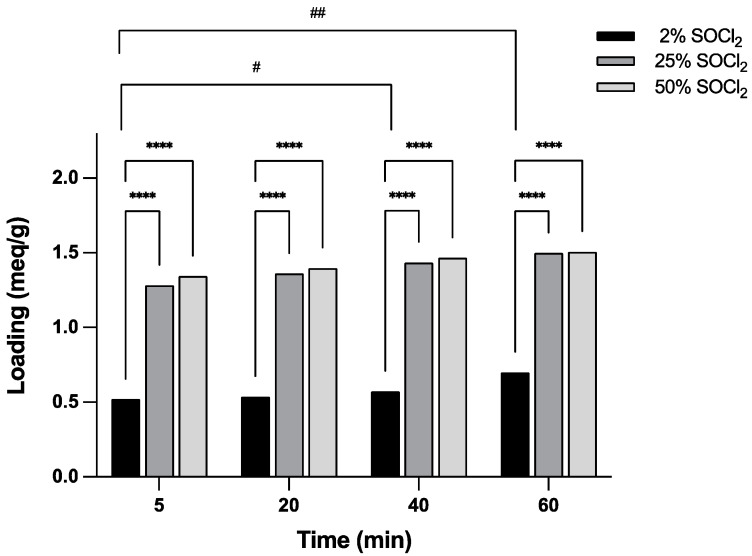
Loading of activated 2-CTC resins obtained under different % SOCl_2_ and activation times. The significant differences between the % of SOCl_2_ used was analyzed by Tukey’s multiple comparison test, are indicated in bold as follows: **** *p* < 0.0001. Significant differences in activation time, analyzed by Tukey´s multiple comparison test, are indicated in bold as follows: ## *p* < 0.01, and # *p* < 0.05.

The effect of the SOCl_2_ concentration and the activation time on resin loading and activation was determined at three concentrations and four different times, as indicated in Table 2 and Figure 2.

### 3.2. Incorporation of the First Amino Acid 

To evaluate the impact of moisture on resin loading, the resin was activated by using 50% of SOCl_2_ in anh. DCM and the addition of H_2_O at different steps:

1. By adding H_2_O alongside the activation solution of 50% SOCl_2_ (second column Table 3)
2. By adding H_2_O into the amino acid coupling solution (third column Table 3)

The process of incorporating the first amino acid followed the procedure outlined in Section 2.3.3, and the results corresponding to the resin loading are presented in Table 3.

### 3.3. Model Tripeptide 

Several samples of resin were chosen to synthesize the model tripeptide Fmoc-Gly-Lys(Boc)-Thr(tBu)-OH. The yield was calculated in terms of resin gain weight, and the % purity was evaluated by integrating the main peak in the corresponding chromatogram (Table 4).

## 4. Discussion

Due to the increased demand of peptides for both research and industrial purposes [10], peptide synthesis is a process that is constantly under review, seeking new procedures and reagents to make it more efficient and greener. However, this is a rather slow process, and in the case of changing solvents and reagents, while new options are being evaluated, many laboratories still rely on the most common reagents and solvents used in the past. In this context, having protocols that optimize its usage is necessary, as is intended with the current study.

In the activation process (Table 2), the reaction proceeded very quickly. With 50% SOCl_2_, a loading of 1.34 mEq/g (84.5%) was achieved within 5 min of reaction time. Extending the reaction time to 60 min brought about only a modest increase of 10.3% in the loading, which reached 94.8%. The most significant differences in resin loading were obtained between the 5- and the 40- or 60-min treatments. In terms of concentration, significant differences occurred between 2% and 25% or 50% SOCl_2_ at each time point, but no significant differences were observed between 25% and 50% SOCl_2_. The use of 2% SOCl_2_ to activate the 2-CTC resin produces a minor increase in loading values, ranging from 30.7% to 44.0% (Table 2). However, these values may be adequate for synthesizing medium-sized peptides consisting of 10 to 20 amino acids. This represents a 25-fold reduction in SOCl_2_ usage compared to 50% SOCl_2_, which is economically important for cost reduction and also environmentally beneficial in reducing pollution.

Of note, the impact of moisture is evident by comparing the resin loading reported by the supplier (1.59 mEq/g) and the resin loading measured before activation (0.35 mEq/g) (Table 3). This comparison indicates a loss of 77.7% of the original resin substitution as a result of moisture.

**Figure 3:**
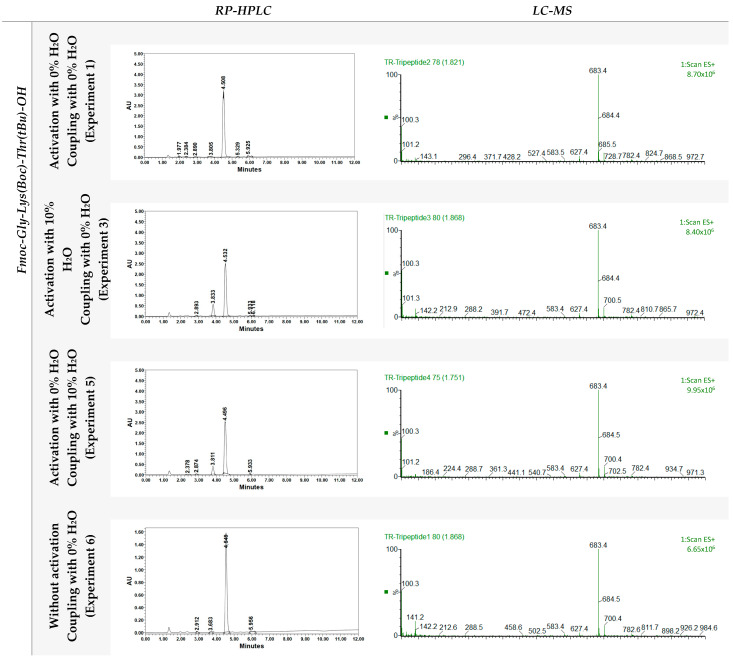
Analysis by reverse-phase high-performance liquid chromatography (RP-HPLC) and liquid chromatography-mass spectrometry (LC-MS) of crude Fmoc-Gly-Lys(Boc)-Thr(tBu)-OH, using a 50 to 100% acetonitrile gradient in 8 min for RP-HPLC and 50 to 100% acetonitrile gradient in 3.5 min for LC-MS.

The effect of moisture was more pronounced in the activation stage than in the coupling stage (Table 3). When 5% H_2_O was added during the resin activation step (Experiment 2), the loading reached only 49.7%. On the other hand, when 5% H_2_O was added during the coupling step (Experiment 4 in Table 3), resin loading reached 65.2%. Increasing the water content to 10% during the activation step (Experiment 3 in Table 3) reduced resin loading to 47.5%, while using 10% water during the coupling step (Experiment 5 in Table 3) resulted in a loading of 53.1%. Although the presence of both 5 and 10% of H_2_O should led to saturated solutions there is difference between the use of both solutions. In comparison, without the addition of water (Experiment 1 in Table 3), a loading of 93.4% was achieved. It is important to highlight that the two synthesis carried out with the resins manipulated in the presence of H_2_O present an impurity at approximately 3.8 min (Figure 3). Although its structure has not been possible to determine, it is a clear indication on how important is to avoid humidity when using 2-CTC resin.

Resins with high loadings are employed in the synthesis of short peptides. In the case of 2-CTC resin, it proves to be exceptionally valuable for constructing just dipeptides or small protected peptides [9] or as a polymeric protecting group [11], as seen in amino acid methylation (work under review). These scenarios highlight situations where achieving high yields is desirable.

In the study by López et al. [12], where N-butylpyrrolidinone is used as the primary solvent, they find that the water content in the solvent is a significant factor for achieving a high resin substitution. When comparing with DCM, they employ a low amount of water (50 ppm), so a reduction in substitution due to the water’s influence is not observed in this case.

In this report, we observe that when utilizing DCM saturated in water, the substitution effect is a significant factor both in the resin activation and in the coupling of the first amino acid, with a more pronounced impact in the activation step. These findings highlight the importance of using anhydrous DCM in both the activation and coupling stages to minimize the adverse effects of moisture on resin loading.

## 5. Conclusions

Moisture is a critical factor that must be controlled when activating 2-CTC resin as it can impair resin loading during the synthetic process.

The resin load was verified after the incorporation of the first amino acid under different conditions. Furthermore, it was confirmed by evaluating the synthesis yield of a test tripeptide. Three concentrations of SOCl_2_ were used in the activation step: 2%, 25%, and 50%. The results were similar for the last two concentrations. Additionally, with only 5 min of reaction, resin loadings of more than 80% were achieved. For the case of 2% SOCl_2_, the resin was activated, and the loading ranged between 0.52 mEq/g (30%) and 0.69 mEq/g (44%), which can be used in the synthesis of short peptides and allows a considerable reduction in the amounts of required reagents.

Optimizing the activation of 2-CTC resin using anh. DCM allowed a reduction in the use of reagents, as well as in reaction time. Furthermore, the feasibility of resin recovery for reuse after activation was demonstrated for the described procedure. These findings were validated through the successful synthesis of a test tripeptide.

## Figures and Tables

**Table 1:** Experiments carried out to determine the effect of moisture on resin loading.

#	H_2_O (%) in 50% *v*/*v* SOCl_2_/anh. DCM	H_2_O (%) in the Coupling Solution
1	0	0
2	5	0
3	10	0
4	0	5
5	0	10
6 *	No activation	0

* # 6 corresponds to the use of the resin directly from the supplier without activation.

**Table 2:** Resin loading calculated at different SOCl_2_ concentrations and different activation times in mEq/g and percentage with respect to the theoretical loading of 1.59 mEq/g.

Loading								
SOCl_2_	Time (min)							
	5		20		40		60	
	mEq/g	%	mEq/g	%	mEq/g	%	mEq/g	%
2%	0.52	30.7	0.54	33.8	0.57	36.0	0.69	44.0
25%	1.28	80.8	1.34	85.7	1.43	90.2	1.49	94.3
50%	1.34	84.5	1.39	87.9	1.47	92.3	1.51	94.8

**Table 3:** Incorporation of the first amino acid. Resin activation with 50% *v*/*v* SOCl_2_/anh. DCM. Loading was measured after the first amino acid coupling.

Experiment	H_2_O (%) in 50% *v*/*v* SOCl_2_/anh. DCM	H_2_O (%) in the Coupling Solution	Loading (mEq/g)	% Loading *
1	0	0	1.49	93.4
2	5	0	0.79	49.7
3	10	0	0.76	47.5
4	0	5	1.04	65.2
5	0	10	0.85	53.1
6	0/No activation	0	0.35	22.3
Theoretical **	-	-	1.59	100

* Resin activation %, considering the theoretical loading as 100%. ** Loading provided by the supplier.

**Table 4:** Yield and characterization of the crude tripeptide Fmoc-Gly-Lys(Boc)-Thr(tBu)-OH synthesized on 2-CTC resin under different moisture conditions.

Experiment	Initial 2-CTC Resin Weight (mg)	Loading (mEq/g)	Theoretical Peptide Weight (mg)	Experimental Peptide Weight (mg)	% Yield	Retention Time (tr)	% Purity
1	198.8	1.49	201.4	180.3	89.5	4.508	95.4
3	202.5	0.76	103.3	89.3	86.5	4.532	84.0
5	199.9	0.85	115.3	102.1	88.6	4.496	87.1
6	201.0	0.35	48.6	35.1	72.3	4.549	97.7

## Data Availability

Data sharing not applicable. No new data were created or analyzed in this study. Data sharing is not applicable to this article.
